# New Aspects of Bilayer Lipid Membranes for the Analysis of Ion Channel Functions

**DOI:** 10.3390/membranes12090863

**Published:** 2022-09-06

**Authors:** Hironori Kageyama, Teng Ma, Madoka Sato, Maki Komiya, Daisuke Tadaki, Ayumi Hirano-Iwata

**Affiliations:** 1 Graduate School of Biomedical Engineering, Tohoku University, 6-6 Aoba, Aramaki, Aoba-ku, Sendai 980-8579, Japan; 2 Research Institute of Electrical Communication, Tohoku University, 2-1-1 Katahira, Aoba-ku, Sendai 980-8577, Japan; 3 Advanced Institute for Materials Research (WPI-AIMR), Tohoku University, 2-2-1 Katahira, Aoba-ku, Sendai 980-8577, Japan

**Keywords:** bilayer lipid membranes, ion channel, lateral voltage

## Abstract

The bilayer lipid membrane (BLM) is the main structural component of cell membranes, in which various membrane proteins are embedded. Artificially formed BLMs have been used as a platform in studies of the functions of membrane proteins, including various ion channels. In this review, we summarize recent advances that have been made on artificial BLM systems for the analysis of ion channel functions. We focus on two BLM-based systems, cell-membrane mimicry and four-terminal BLM systems. As a cell-membrane-mimicking system, an efficient screening platform for the evaluation of drug side effects that act on a cell-free synthesized channel has been developed, and its prospects for use in personalized medicine will be discussed. In the four-terminal BLMs, we introduce “lateral voltage” to BLM systems as a novel input to regulate channel activities, in addition to the traditional transmembrane voltages. Such state-of-the-art technologies and new system setups are predicted to pave the way for a variety of applications, in both fundamental physiology and in drug discovery.

## 1. Introduction

The cell is surrounded by a membrane composed of bilayer lipid membranes (BLMs) and membrane proteins. The BLM has an ultrahigh resistance, higher than 1 TΩ [1], serving as a barrier for the permeation of ions across the cell membrane. Among the various membrane proteins, ion channels regulate transmembrane ion flow and play a key role in various physiological processes, including in the generation of the action potential, nerve transmission, and heartbeat [2]. Owing to their physiological importance, ion channels are also major targets for drug design [3]. In contrast to recent drastic progress in the structural analysis of ion channels due to the advent of the cryo-electron microscope [4,5], progress in the functional analysis of ion channels has remained moderate, with the patch-clamp method (awarded the Nobel Prize in 1991) still being the gold standard. There is a great demand for a novel functional analysis system that could provide new insights into ion channels in terms of physiological and pharmaceutical aspects.

The reconstitution of ion channel proteins in artificially formed BLMs is another approach for recording ion channel activities [6]. BLMs with channels imbedded in them can be regarded as cell-membrane-mimicking systems that allow the buffer and membrane conditions to be controlled at a higher level than actual cell membranes. Combined with the rapidly evolving synthetic biology using cell-free protein expression technologies [7,8], researchers can, in principle, design BLMs that contain only one channel genotype, leading to the development of a next-generation drug-screening platform. In addition, BLM systems also allow innovative experimental settings that are impossible with cell membranes. For example, when we place additional electrodes in the BLM interior in addition to the two conventional electrodes for transmembrane voltages, the additional electrodes can introduce a new input, namely a lateral voltage, to the BLM system [9,10]. The introduction of a lateral voltage has the potential to revolutionize the functional analysis of ion channels as the evolution from diodes (single input) to transistors (double inputs) accelerated the progress in the semiconductor technology [11].

**Figure 1:**
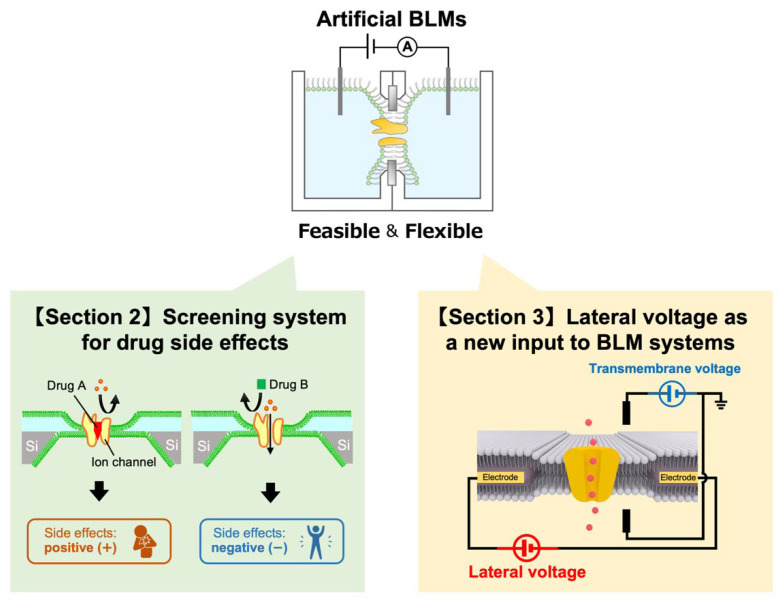
Two systems for ion channel analysis based on bilayer lipid membranes (BLMs).

In this review, we summarize recent advances made by our research group on the construction of a cell-membrane mimicry system and lateral voltage systems for the functional analysis of ion channels (Figure 1). As a cell-membrane mimicry system, we reconstituted human ion channels into BLMs to mimic the human cell membrane. The BLMs with cell-free synthesized ion channels imbedded in them were then used to evaluate potential drug effects, and their prospects for use in personalized medicine are also discussed [6,12]. We also constructed a new BLM platform in which a lateral voltage was applied to the BLM interior. The effect of the lateral voltage on the activities of a voltage-dependent sodium channel was investigated and confirmed.

## 2. BLM-Based Screening Systems for Examining Drug Side Effects and Their Prospect for Personalized Medicine

Ion channel proteins are major targets of drug design for the treatment of various diseases, such as neural disorders, cardiac arrhythmia, cancer, respiratory disorders, and pain [13,14,15,16]. However, unintentional interactions between drugs and ion channels also result in severe side effects. A representative example that received considerable attention is the human *ether-a-go-go*-related gene (hERG) channel, a voltage-dependent potassium channel in the heart muscle [17]. Diverse groups of drugs have caused an unintentional blockade of the hERG channels, which sometimes induces life-threatening arrhythmias. Many of these drugs have been withdrawn due to their serious side effects on the hERG channel [17,18]. In addition, a possible relationship has been proposed between such drug-induced arrhythmia and the hERG channel genotypes [19]. It is now highly important to develop a novel screening system in which we could assess the potential risks of drug side effects for respective patients. For this purpose, a combination of cell-free expression systems and a reconstitution of BLM systems would be promising. Using cell-free expression systems, we can synthesize target ion channel proteins in vitro from a DNA template within several hours [7]. Compared with cell expression systems, such as HEK293 cell systems, it is possible to directly generate target proteins of various channel genotypes without labor-intensive cell culture, transfection, and protein isolation.

**Figure 2:**
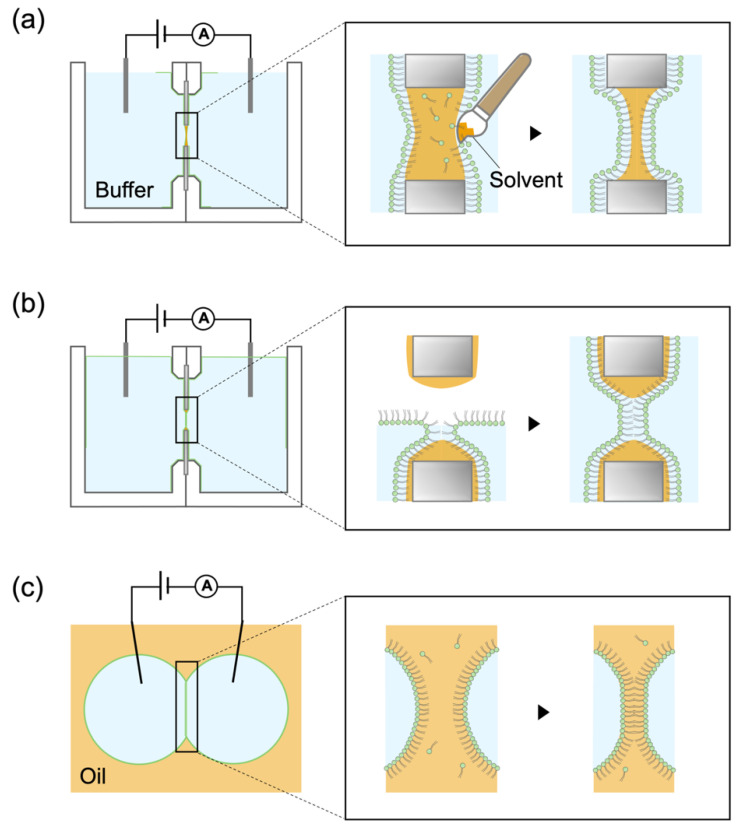
Schemes showing three methods for the formation of BLMs (not to scale): (**a**) painting method [20]; (**b**) monolayer folding method [22]; (**c**) droplet interface bilayer (DIB) method [27]. In the DIB method, BLMs are formed by bringing two lipid-coated aqueous droplets into contact in an oil phase. Reprinted with modification from ref. [6]. Copyright 2020 WILEY.

Another key factor in the BLM-based drug-screening platform is how to consider the effect of oil (nonvolatile organic solvents), such as decane, hexadecane, squalane, and squalene. Since the first report of the formation of a BLM by Mueller et al. in 1962 [20], BLMs have been formed via the self-assembly of amphiphilic lipids in an aqueous environment [6,21,22]. A nonvolatile organic solvent is often effectively utilized to provide an oil–water interface to assist in the formation of a stable BLM [22,23,24,25,26] (Figure 2). However, when applying the BLM systems to a drug screening platform, the presence of oil might cause an increase in the concentration of hydrophobic drugs in the oil and add further complexity to determining the concentration of a target drug in an aqueous phase [6]. Therefore, a solvent-free BLM would be preferable as a drug-screening platform, though the BLM stability could be further weakened in the absence of a solvent.

### 2.1. Fabrication of Tapered Apertures to Form Stable Solvent-Free BLMs

**Figure 3:**
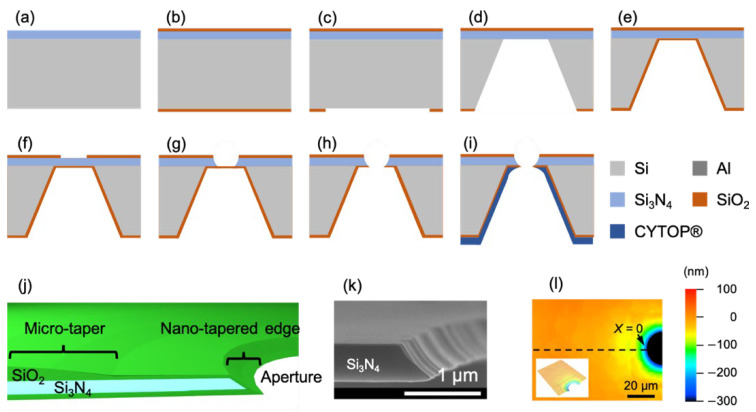
Fabrication process (**a**–**i**), structural scheme (**j**), scanning electron microscopic image (**k**), and height profile (**l**) of the Si chip used to produce BLM. After the aperture was formed in the Si chip (**h**), fluoropolymer CYTOP^®^ (CTL-809M) was coated on an entire chip to reduce the capacitance of the chip. Reprinted with permission from Refs. [11,12].

BLMs are commonly formed either in micro- and nano-apertures that are fabricated in insulating partitions [21,22,24,28,29] or at the interface between water droplets and lipid-containing oils [25,26,27,30] (Figure 2). BLM formation in an aperture still requires oil to seal the gap between the ultrathin BLMs and the partition. If we can reduce the gap and form a smooth connection between the BLM and the partition, it is possible to produce stable BLMs without the need for oil. To achieve this, we adopted the silicon (Si) micro-fabrication technologies to produce circular micro-apertures (φ: 20–60 µm) whose edges were smoothly tapered [12,31,32]. The micro-aperture was fabricated on a silicon nitride (SiN) layer on a Si chip by standard photolithography methods and a wet-etching method (Figure 3a–i). The edge of the fabricated aperture tapered gradually (Figure 3j). Solvent-free BLMs were stably formed in the tapered aperture by the folding method (Figure 2b). The resulting product had an average lifetime of 16 h [33] and a maximum lifetime of 20 days [12]. In addition to static stability, the BLMs were also mechanically stable, exhibiting tolerance to various mechanical shocks, including repetitive exchanges of the solution, the movement of water around the BLM, and the application of a centrifugal force [12,21,33,34].

### 2.2. Applications to a Drug-Screening Platform

**Figure 4:**
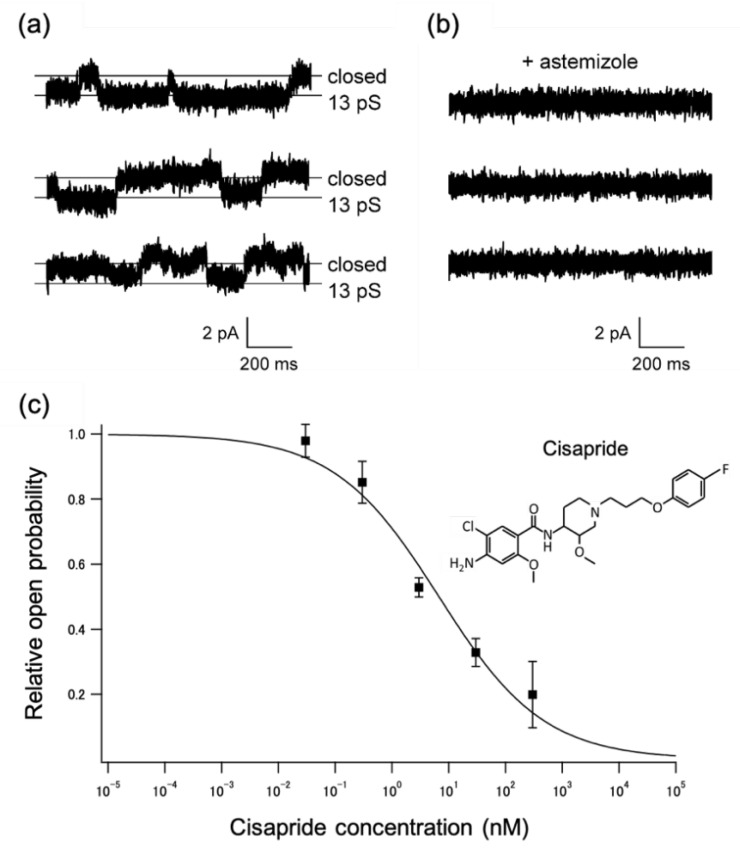
Examples of single-channel currents observed with BLMs containing cell-free synthesized wild-type hERG channels. All the currents were recorded at −100 mV after a 300 ms prepulse of +50 mV. (**a**) Typical single-channel currents; (**b**) current traces after the addition of astemizole (1 µM); (**c**) relationship between cisapride concentration and relative open probability of the cell-free synthesized hERG channels. Lipid composition was L-α-phosphatidylcholine (PC):L-α-phosphatidylethanolamine (PE):cholesterol = 7:1:2 (weight ratio). A symmetric buffer containing 120 mM KCl and 10 mM HEPES (pH 7.2 in KOH) was used in these experiments. Current signals were filtered at 1 kHz with a low-pass Bessel filter and digitized at 10 kHz. The current data were offline filtered at a cut-off frequency of 0.7 kHz. Proteoliposomes containing the hERG channels were added to one side (*cis*) of the BLMs, and drugs were added to the other side (*trans*). Applied potentials were defined as the potential at the *cis* side relative to the *trans* side held at the ground. Reprinted with permission from refs. [6,12]. Copyright 2020 WILEY.

To illustrate the potential of using BLMs containing ion channels as a drug-screening platform, we incorporated hERG channels into stable BLM systems. The hERG channel was synthesized using a wheat germ-cell-free expression system and was incorporated into the solvent-free BLMs via fusion between liposomes containing the hERG channel and the BLMs [12]. The incorporation process was facilitated by applying a centrifugal force to cause the proteoliposomes to concentrate near the BLMs [34]. Figure 4 shows examples of these hERG channel activities. Clear rectangular currents that correspond to single-channel activities were recorded. The observed conductance (13 ± 0.2 pS, *n* = 12) was similar to previously reported values (10–13 pS) [35,36,37]. The channel activities were blocked by astemizole, an antihistamine that may cause an arrhythmia through the unintentional blockade of hERG channels [18]. The cell-free synthesized hERG channels that were imbedded in the present BLM systems exhibited similar channel properties, such as single-channel conductance and sensitivity to astemizole, to those reported with the hERG channels isolated from the cells overexpressing them [21,38].

**Figure 5:**
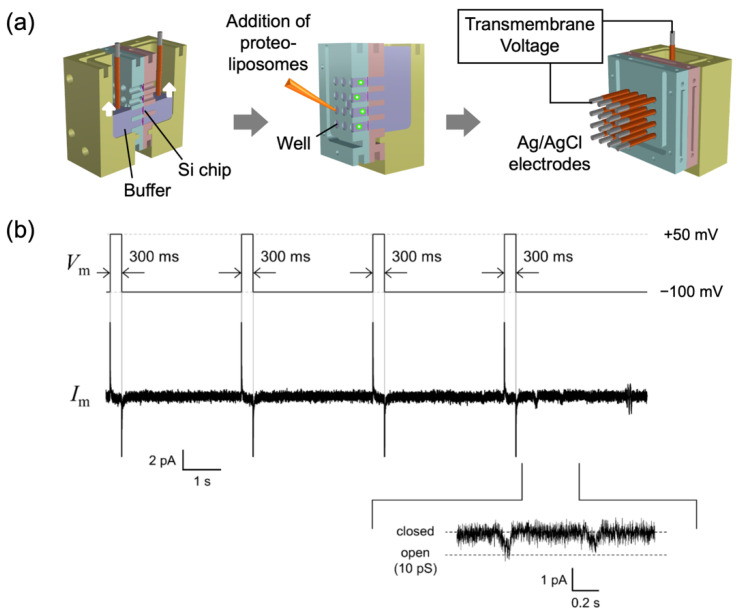
(**a**) Schematic images of a 16-well BLM analysis system; (**b**) an example of hERG single-channel current elicited by a repetitive voltage-step protocol. Signals were recorded with a 1 kHz low-pass filter at a sampling frequency of 20 kHz. The current traces were offline filtered at a cut-off frequency of 0.7 kHz. Reprinted with permission from Ref. [44].

We next investigated the relationship between the drug concentration and hERG channel activities to determine the half-maximal inhibitory concentration (IC_50_), an indicator of the inhibiting behavior of a drug. Since the drug concentration in the aqueous phase is important, the drugs that were known to adhere easily to the surface of measuring containers, including astemizole, were unsuitable for this experiment [39]. The gastroprokinetic agent cisapride was used as a representative drug because of its well-known inhibitory effect on hERG channels with a low propensity to adhere to the surfaces [18,39,40]. As shown in Figure 4c, the open probability of the hERG channels decreased with an increase in extracellular cisapride concentration. The calculated IC_50_ value based on the concentration dependence was 6.7 ± 2.0 nM (n = 3) [6], which is similar to those (6.5–20.5 nM) reported in patch-clamp studies [40,41,42]. These results demonstrate that the present BLM system is a useful drug-screening platform for ion channels. Through the extension of this approach to various drugs and hERG genotypes, it is possible to construct a database for hERG genotypes vs. potential drug side effects [6]. To further speed up the screening process, the use of arrays of BLMs is an intuitive and useful approach for obtaining parallel recordings of ion channel activities [43]. We also developed a BLM array system, in which 16 wells for the BLM formation were aligned in parallel [44] (Figure 5). Using the hERG channel produced by the HEK 293 cell expression system, we verified the simultaneous formation of multiple BLMs and succeeded in parallel recording the channel activities.

## 3. Lateral Voltage as a New Input to BLM Systems

**Figure 6:**
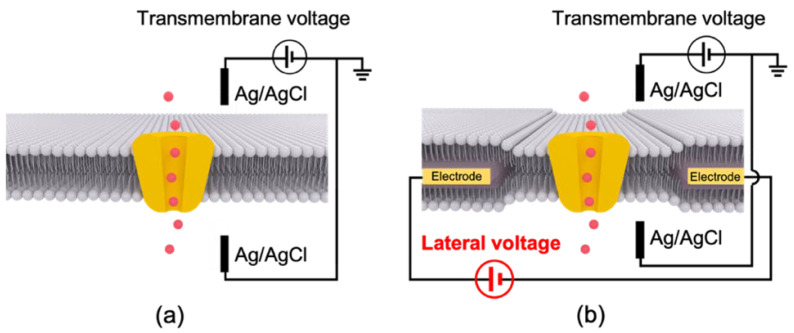
Conceptual schemes of (**a**) conventional voltage-clamp method and (**b**) proposed four-terminal BLM system with an additional lateral voltage as a system input (not to scale). Note that the actual BLMs are formed vertically by the folding method (see Figure 2b).

Functional evaluations of ion channels are commonly performed based on the current measurement under the control of a transmembrane voltage (Figure 6a), the potential difference between the solutions on both sides of the membranes, including both biological cell membranes, and artificially formed BLMs. In both cases, the transmembrane voltage is applied via two electrodes immersed in two buffer solutions that sandwich the membranes. This configuration has not changed substantially since the discovery of the voltage-clamp method, irrespective of extensive progress and changes in target samples from squid giant axons [45,46] to neuronal cells [47], eukaryotic cells that can overexpress the ion channel of interest [48,49], and artificial BLMs containing cell-free synthesized channels [12,30,50,51,52,53].

We recently introduced another novel voltage to a BLM recording system, a lateral voltage that is parallel to the membrane plane [11] (Figure 6b). In addition to the conventional two Ag/AgCl electrodes for transmembrane voltage, two metal electrodes were wired around a micro-aperture on the partition material that was used for supporting the BLMs. Since the partition with the metal electrodes was placed between two lipid monolayers, the formation of the BLMs by folding up the two monolayers in the micro-aperture led to the insertion of the electrodes between the two leaflets of the BLMs. The lateral voltage can be applied to the interior of the BLMs through these electrodes. Since this is a new configuration for BLM experiments, two types of membrane supports, Si chips and conventional Teflon films, were explored. We investigated the BLM formation with these electrode-wired supports and compared the noise properties between the two materials. The effects of the lateral voltage on the activities of voltage-gated ion channels that had been incorporated in the BLMs were also evaluated.

### 3.1. Fabrication of Electrode-Wired Membrane Support for the Application of Lateral Voltage

**Figure 7:**
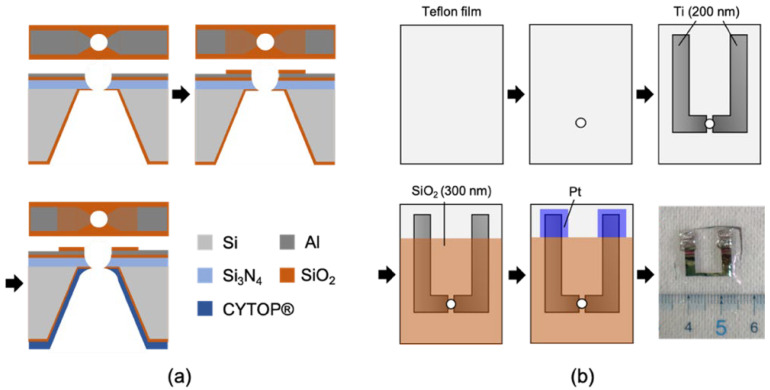
The fabrication process of (**a**) Si chip and (**b**) Teflon film equipped with metal electrodes for lateral voltage application. Reprinted with modification from Refs. [10,11]. Copyright 2019 American Chemistry Society and 2022 The Royal Society of Chemistry.

We first attempted to wire the electrodes on the Si chips, as described in Section 2 [10]. By using the Si chip (h) in Figure 3 as the starting material, aluminum (Al) electrodes were deposited on the chip by thermal evaporation (Figure 7a). A SiO_2_ layer was sputtered at the center part of the chip to function as a passivation layer to protect the electrodes from corrosion. Finally, a fluoropolymer CYTOP^®^ was formed on the Si side of the chip.

Teflon films have commonly been used as a support in forming BLMs [22,54,55]. We also used Teflon as a base material for wiring electrodes for lateral voltages. Micro-apertures (φ = 100–150 µm) were first formed on a Teflon film by an electric spark. Titanium (Ti) electrodes were then deposited on the top of the film by electron beam (EB) evaporation (thickness: 200 nm) (Figure 7b). Similar to the Si chip process, we formed a protective SiO_2_ layer (thickness: 300 nm) on the Ti electrodes. We then deposited a platinum (Pt) layer on the exposed Ti electrodes to prevent the oxidation of the Ti surfaces. The fabricated Si chip and Teflon film were silanized with (tridecafluoro-1,1,2,2-tetrahydrooctyl) dimethylchlorosilane (PFDS) at room temperature [12,33].

### 3.2. BLM Formation in Electrode-Wired Membrane Supports

The formation of BLMs was first investigated using the electrode-wired Si chip and the Teflon film. We confirmed that there was no leakage current between the two metal electrodes on the membrane support. The BLM formation was examined by the folding method (Figure 2b). In the case of the Teflon film, the aperture was pre-treated with hexadecane to seal the gap between the nm-thick BLM and the μm-thick Teflon film. After folding two lipid monolayers across the apertures fabricated in both types of membrane supports, the resistance between the two Ag/AgCl electrodes was found to exceed 200 GΩ, thus confirming that highly resistive BLMs were formed. No significant changes in the BLM resistance and capacitance were observed between the membrane supports with and without electrode wiring. Therefore, the presence of electrodes and SiO_2_ layers around the apertures had no measurable effects on the formation of the BLMs.

**Figure 8:**
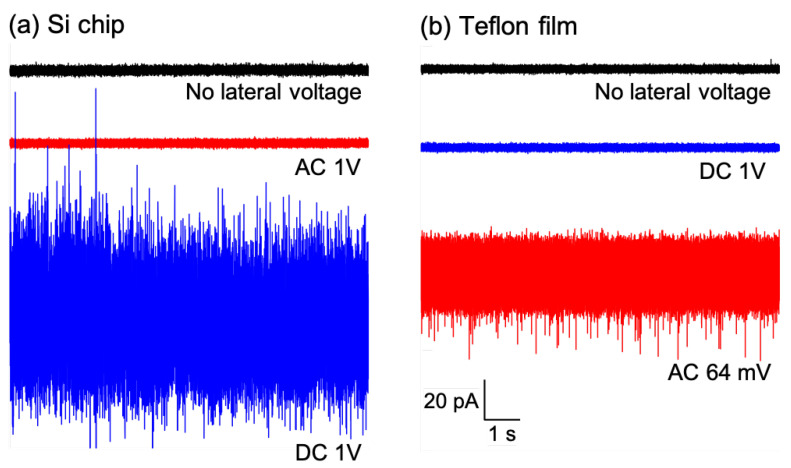
Transmembrane current noise from Si chip (**a**)- and Teflon film (**b**)-based BLM systems. Noise levels without lateral voltage (black), with AC lateral voltage (red), and with DC lateral voltage (blue). Reprinted with modification from Ref. [11]. Copyright 2022 The Royal Society of Chemistry.

We then investigated the effects of lateral voltage (DC and AC voltages) on the stability and transmembrane current noise with the BLMs that had been formed in Si chips and Teflon films. Applying an AC lateral voltage to the BLMs in the Si chip induced no significant changes in the stability and noise level [10,11]. Under a lateral voltage of AC 1 V (peak-to-peak), the resistance was still high (>200 GΩ) and the noise level was low (∼3 pA in peak-to-peak), which was comparable to that of the BLMs without a lateral voltage (Figure 8a). When we applied a DC lateral voltage (1 V) to the BLMs in the Si chip, the membrane resistance decreased to ~100 GΩ, while the noise level increased significantly (3 pA to over 80 pA), thus making it unsuited for use in collecting single-channel recordings (Figure 8a). On the other hand, in the Teflon-film-based system, the BLM maintained a high resistance over 200 GΩ and a low noise level (∼3 pA) under the application of a DC lateral voltage (1 V) [11] (Figure 8b). In contrast, even a small AC lateral voltage (64 mV in peak-to-peak) caused a dramatic increase in the noise (>40 pA), and a higher AC voltage (0.5 V) caused the BLM to rupture. Thus, the BLMs in the Teflon film and the Si chip showed opposite noise characteristics for the application of the DC and AC lateral voltages; the Teflon film is suitable for the application of DC lateral voltages, while the Si chip is suitable for the application of AC lateral voltages. For studying the effects of the lateral voltage on the activities of ion channels, we started with a combination of the Teflon film system and a DC lateral voltage that is simpler with fewer variable factors than AC voltages.

### 3.3. Effect of a Lateral Voltage on the Activities of Ion Channels Embedded in BLMs

As described in Section 2, ion channel functions, especially those of voltage-gated channels, have been analyzed in terms of transmembrane voltage [18,56]. For example, the activities of hERG channels are commonly elicited by step pulses of transmembrane voltages (Figure 5b). However, it was observed that some ion channels rapidly lost their activities to show no opening events. After passing through such a non-conducting state, they never returned to show channel activities. If another input, namely a lateral voltage, were to be applied to the BLM system, the lateral voltage may switch the conducting activities of the channel. We examined this possibility by using the human voltage-gated sodium channel (Na_V_1.5) as an illustrative example. The Na_V_1.5 channel plays a crucial role in the generation of an action potential in cardiomyocytes [57,58].

**Figure 9:**
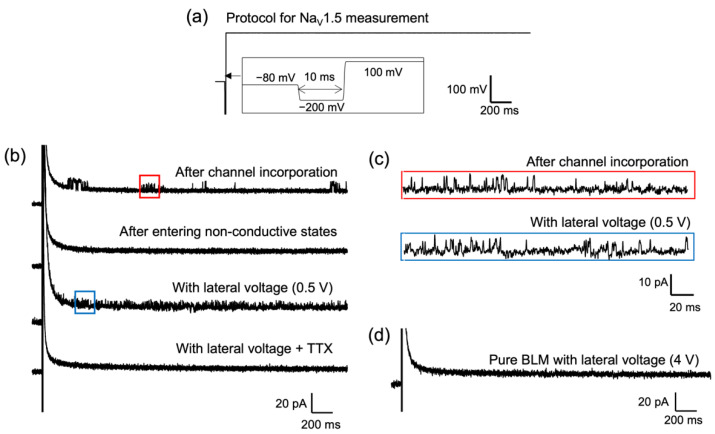
(**a**) Transmembrane voltage protocol for recording Na_V_1.5 channel currents; (**b**) sequentially measured transmembrane currents after incorporation of the channel; (**c**) detailed channel activities marked by red and blue squares in (**b**); (**d**) the transmembrane current of a BLM without channels under a lateral voltage of DC 4 V. Current signals were filtered at 2 kHz with a low-pass Bessel filter and digitized at 25 kHz. Reprinted with modification from Ref. [11]. Copyright 2022 The Royal Society of Chemistry.

The BLMs were formed in Teflon films on which two Ti electrodes were patterned. The proteoliposomes were prepared from Na_V_1.5-transfected HEK293T cells and were fused to the BLMs for the incorporation of the channel. When a transmembrane voltage protocol that is commonly used to elicit Na_V_1.5 activities [59] (Figure 9a) was applied to the BLMs, sporadic channel currents were observed (Figure 9b). However, repeated sweeps of the voltage protocol led to a disappearance of channel activities. The channel activities failed to be recovered on the further repetition of the transmembrane voltage protocol. When we additionally applied a lateral voltage (DC 0.5 V), the channel activities were recovered and drastically enhanced. As shown in Figure 9c, the channel activities under the application of the lateral voltage were similar to that of the initial channel activities. These channel activities were completely blocked by the addition of tetrodotoxin (TTX), a specific inhibitor of sodium channels (Figure 9b). No significant current responses were elicited by the same transmembrane protocol when a lateral voltage of DC 4 V was applied to pure BLMs without ion channels. These results suggest that applying a lateral voltage can allow the activities of the Na_V_1.5 channel that were in non-conductive states to be recovered. The lateral voltage can be a useful additional input for the functional analysis of ion channels, whose activities would have been concealed under the traditional (transmembrane) voltage-clamp conditions.

## 4. Summary and Perspectives

Since the first report of artificially formed BLMs in 1962, BLMs with reconstituted ion channels are now considered to be a complementary system for the patch-clamp method. Although the mechanical fragility of BLMs has been a major obstacle to the use of BLM reconstitution systems, the fabrication of micro-apertures with nano-tapered edge structures has greatly improved the mechanical stability and durability of BLMs. Recent progress in cell-free protein synthesis has also opened new possibilities for using BLM systems as functional evaluation and drug-screening systems for synthesized ion channels. Through the combination of stable BLMs and cell-free expression systems, we succeeded in recording single-channel currents of a wild-type hERG and quantifying the inhibitory effect of a drug on the channel. Extending this approach to various drugs and hERG genotypes will provide a database of hERG genotypes vs. potential drug side effects, which could then be utilized to diagnose and select an appropriate medication for individual patients. We hope that advancing our BLM reconstitution systems will help to achieve such personalized medicine and health care.

We also explored a new possibility of the BLM systems through the introduction of a “lateral voltage” as an input, in addition to the conventional transmembrane voltages. For the application of the lateral voltage to the interior of the BLMs, we wired two metal electrodes around a micro-aperture on a membrane support. After BLM formation across the aperture, the two metal electrodes were automatically inserted inside the two lipid leaflets. It was demonstrated that the lateral voltage application can allow the activities of the cardiac sodium channel (Na_V_1.5), which was in a non-conductive state under the control of conventional transmembrane voltages, to be recovered. Although further investigation will be necessary to reveal the mechanism responsible for regulating channel activities by the lateral voltages, including the distribution of the lateral electric field inside BLMs, the lateral voltage may be a useful new input for the functional analysis of channels that have been difficult to assess with the conventional method.

## Data Availability

Not applicable.
